# Dual Coding or Cognitive Load? Exploring the Effect of Multimodal Input on English as a Foreign Language Learners’ Vocabulary Learning

**DOI:** 10.3389/fpsyg.2022.834706

**Published:** 2022-03-11

**Authors:** Wenwen Li, Jia Yu, Zina Zhang, Xiaobin Liu

**Affiliations:** School of Foreign Studies, South China Normal University, Guangzhou, China

**Keywords:** multimodal input, monomodal input, EFL vocabulary, cognitive load, dual coding

## Abstract

In the era of eLearning 4.0, many researchers have suggested that multimodal input helps to enhance second language (L2) vocabulary learning. However, previous studies on the effects of multimodal teaching have failed to yield definitive conclusions. Furthermore, only few studies on the multimodal input of vocabulary learning have aimed at junior high school students and have focused on explicit vocabulary instruction in class. To explore the effects of multimodal input on English as a foreign language (EFL) learners’ vocabulary learning and summarize effective methods, this study adopts a mixed-method approach. Based on dual coding theory and cognitive load theory, the teaching materials in this study were designed using the resources provided by the multimodal corpus iWeb and other websites. A total of 60 junior high school students who learned EFL and had a similar English proficiency level were divided into an experimental group (EG) and a control group (CG). Target words were selected through questionnaire I. During the experiment, the CG learned from monomodal materials while the EG received multimodal input, and an immediate post-test was delivered to the two groups. Questionnaire II was distributed in the EG, and five students of the EG were randomly selected for an interview. One week later, a delayed post-test was conducted on the EG and CG. The results showed that the EG performed better in the post-test but did worse than the CG in the delayed post-test. The results of the questionnaire and the interview suggest that students held both positive and negative attitudes toward the multimodal input approach in vocabulary learning. The study concludes with some implications for choosing a multimodal input approach in vocabulary learning, along with a number of suggestions on how to optimize its positive influence and minimize its negative effects.

## Introduction

The importance of learning vocabulary has become widely recognized (Schmitt, 2000; Nation, 2001; Barcroft, 2004; Smidt and Hegelheimer, 2004). However, it is difficult for foreign language learners to obtain the meaning of vocabulary items and relative meanings acquired in a given context of use. It is also difficult for these learners to compensate for this difficulty, as they generally do not have the opportunity to get recurring interaction in the target language that can facilitate retention (Zandieh and Jafarigohar, 2012). Therefore, it is necessary for teachers to offer students an alternative that can improve efficiency and deepen vocabulary learning as well as learning in context.

Under the context of eLearning 4.0, new media and new technology expand the possibilities for vocabulary learning. The emergence of multimodal corpora and application of multimodality theory in vocabulary teaching have been gradually recognized and popularized. However, previous studies on the effects of multimodal teaching have drawn inconsistent conclusions. Although some studies have proved the superiority of the multimodal approach over single-mode (Chun and Plass, 1996a,b; Al-Seghayer, 2001; Ramezanali and Faez, 2019), some other studies (Boers et al., 2017) have reported no benefit of additional modes and no significant difference in L2 vocabulary learning.

Therefore, exploring the effects of multimodal input on junior high school students’ vocabulary learning and summarizing an effective vocabulary teaching method are a promising research direction. The primary purpose of this article is to explore the effects of multimodal input used in explicit instruction in EFL classes by employing the multimodal corpus iWeb. In addition, this study investigates students’ perceptions of multimodal input on English vocabulary instruction. By adopting a mixed-method approach to verify the effects of the multimodal input approach on EFL vocabulary learning in high school, this study provides some referential value for high school EFL vocabulary learning and helps to improve vocabulary teaching approaches.

## Literature Review

### Multimodal Input and English as a Second Language Vocabulary Learning

Dual coding theory, concerned at a fundamental level with the nature of symbolic systems, assumes that memory and cognition are served by two separate systems, one specialized for dealing with verbal information and the other for non-verbal information (Paivio, 1990). From the perspective of dual coding theory, multimodal input plays an important role in English as a second language (ESL) vocabulary teaching and learning.

Supported by modern technology, multimodal input has become available for vocabulary instruction and has gained great attention in eLearning 4.0 (Bujang et al., 2020; Abdelghani et al., 2021). As multimodal materials convey different types of information through both of the two channels, many empirical studies based on multimodal input have employed not only language and still pictures but also audio clips and animations to explore their effects on vocabulary teaching and learning (Bisson et al., 2015; Khezrlou et al., 2017; Alzahrani and Roberts, 2021; Muñoz et al., 2021; Perez, 2022). Lin and Yu (2017) compared the effects on vocabulary learning of eighth grade students under four input conditions: text only, text plus picture, text plus sound, and text and sound plus picture. Their findings showed that the input of text and sound achieved the best scores in the immediate post-test, and that the input of text and sound plus picture achieved the best scores in the delayed post-test. Ramezanali and Faez (2019) compared the effects of monomodal annotation and multimodal annotation on word acquisition. This study designed three experimental groups and one control group, and its outcome testified that multimodal input outperformed monomodal input, and that multimodality of translation and video produced better results than multimodality of translation and audio.

Although the above studies have proved that multimodal input is better than monomodal input in terms of vocabulary acquisition, there are also many studies that have resulted in opposite outcome, showing that adding another modality, such as pictures, audio pronunciation, video, and L2 definition, cannot guarantee better score in learners’ vocabulary acquisition (e.g., Acha, 2009; Boers et al., 2017). One possible reason is suggested by cognitive load theory: low-ability learners might have to allocate more of their cognitive resources to processing visual and verbal information than high-ability learners (Plass et al., 2003). Yeh and Wang (2003), for instance, used a between-participants design, and designed three input conditions, which were text only, text plus picture, and text plus picture and audio pronunciation of the word. Vocabulary learning was examined through a mixture of word association and multiple choice and cloze tests. The authors concluded from the overall post-test scores that the text plus picture input was the most effective. However, they also pointed out that this result might be misleading, as there was no significant difference with the scores obtained under the text-only input. Furthermore, the input enhanced with audio pronunciation yielded the poorest post-test scores, arguably indicating that more is not always better. This may be because both text and audio pronunciation are types of verbal information, and presenting two inputs of the same mode caused extra processing load. Yeh and Wang (2003) stressed that the combination of verbal and pictorial glosses can increase target vocabulary retention more than integrating the three gloss types of word, picture, sound, and only one gloss type of text.

### Multimodal Corpus and Vocabulary Learning

The iWeb (“Intelligent Web”) corpus was created by Mark Davies in mid-2018. It contains about 14 billion words including advanced searches of the top 60,000 words that are not available in other large corpora (Davies and Kim, 2019). Learners can search for a wealth of information on each of these words including its definitions, links to images and audio, translations, related topics, word clusters, collocates, and much more. Khan (2019) used iWeb to improve advanced non-native students’ ability in using four-word lexical bundles in academic writing. In this study, students were asked to focus on four-word clusters and the actual usage of these sequences under the section Concordance Lines. The study concluded that it was possible to help students speak and write like expert users of the English language using the iWeb corpus.

Previous studies on multimodal input in vocabulary learning have mainly focused on college students (Wang and Chen (2018); Diao and Hu (2021), adult learners (Boers et al., 2017), and primary school students (Tragant et al., 2016). Only few studies have worked with junior high school students (Lin and Yu, 2017). Furthermore, majority of studies on multimodal input in vocabulary instruction available so far are focused on incidental vocabulary acquisition, in which the recalling of vocabulary meaning is a by-product of reading or listening, with only two exceptions (Lin and Yu, 2017; Diao and Hu, 2021). It is, therefore, important to verify the effectiveness of multimodal input in explicit vocabulary instruction in class. The materials previous authors used were mostly textbooks, documentaries, or videos; only few studies have used multimodal online corpora (e.g., iWeb) This attests to a certain distance between these studies and the reality of current-day language learning, since online corpora are one of the main information sources for generation Z when it comes to eLearning 4.0. The goal of this study is to address this gap. The study aims to examine the effects of multimodal input and monomodal input on vocabulary learning based on evidence from post-tests, questionnaires, and interviews.

## Materials and Methods

### This Study

This study examined the effectiveness of a multimodal input approach compared with a monomodal input approach in L2 learners’ vocabulary learning and retention. The following research questions guided the study:

RQ1: Which teaching approach, multimodal input of verbal and visual materials, or monomodal input of only verbal materials is more likely to promote students’ acquisition of meaning of target vocabulary?

RQ2: In which aspects does the multimodal input of verbal and visual materials affect students’ acquisition of meaning of target vocabulary?

RQ3: What are L2 learners’ attitudes toward the multimodal input approach?

A mixed-method approach was adopted to answer the research questions. The independent variable was the mode of vocabulary input, including monomodal and multimodal inputs. The dependent variable was students’ scores on vocabulary meaning retrieval tests. The participants were divided into two groups: The control group (CG) and the experimental group (EG). The CG received monomodal input containing only verbal materials, while the EG received multimodal input that comprised of verbal and visual materials selected from iWeb. Vocabulary meaning retrieval tests were distributed to explore students’ learning effects, while questionnaire II and interview were conducted to investigate their attitudes toward multimodal input.

### Participants

The research participants of this study, whose native language was Chinese, were Grade Nine students from two self-paced classes in a junior high school in Shenzhen, Guangdong province. Based on final examination scores of grade eight and the level test conducted at the beginning of grade nine, students whose scores were on the top eight of each class were selected for the two self-paced classes, so students of the two classes had relatively good performance in their study. To ensure that the two groups had similar levels of English proficiency, their midterm examination in English of that semester was analyzed. The English midterm exam was organized by the school, and all students had to participate in it. The exam included listening, reading, and writing items that tested students’ English proficiency. There were multiple-choice questions that tested listening and reading comprehension, fill-in-the-blank questions that examined grammar knowledge, and a writing item that required students to write an English essay. Results of an independent samples *t*-test showed no significant difference between the English proficiency of the two groups (*t* = −0.091, *p* = 0.928 > 0.05). The participants were naturally classified into the control group (CG) and the experimental group (EG) based on their class unit. A total of 32 students were treated as CG, and 28 students were regarded as EG according to their familiarity with the target words, as they declared that they did not learn after they filled out questionnaire I, which was designed by the authors to see the participants’ knowledge of the test items. Table 1 shows information about the participants.

**TABLE 1:** Information about the participants.

	Male	Female	Total
EG	15	13	28
CG	17	15	32

### Teaching Materials

Target words were sorted out using questionnaire I. A total of 48 words were initially chosen from TEENS Junior (*teens.i21st.cn*), which was specifically designed for junior high school students as a complementary material to the textbook in use and was chosen by the English teacher of the two classes. After questionnaire I was distributed, eight words were picked out on the basis that all the participants declared that they had never encountered these words.

**FIGURE 1:**
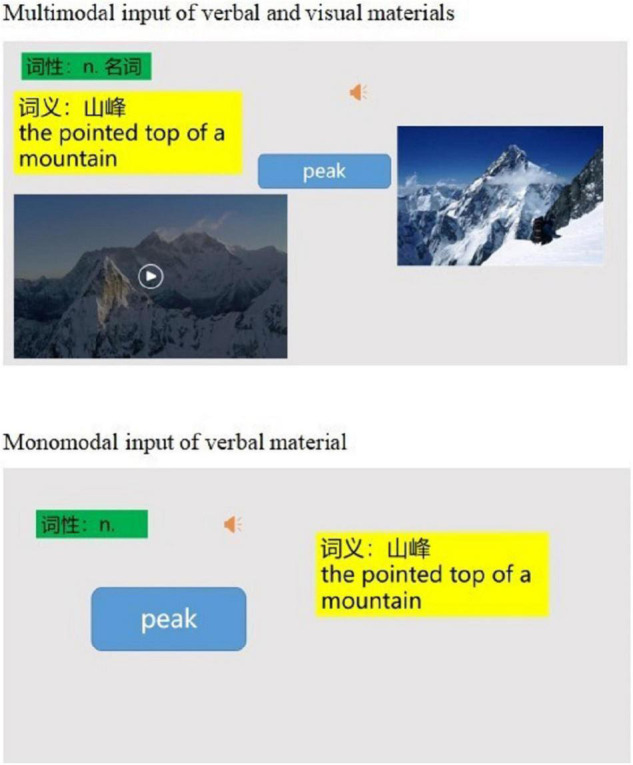
The two experimental treatments in this study illustrated with the target word peak.

Microsoft PowerPoint was used to design the courseware consisting of one slide per target word. To illustrate the interface of the slide, Figure 1 presents a labeled screenshot of the target word *peak* shown in the slide that includes all multimodal verbal and visual representations. The verbal information of the target words is the shared information shown to both the CG and the EG (i.e., L1 translation, L2 definition, written form, and audio pronunciation). The picture and video are visual information designed only for the EG.

The pictures and videos that explain the meaning of the target words were selected by searching iWeb. Then, the multimodal materials were organized and presented in slides. During classroom teaching, the written form of the target words, audios, L2 translations, L1 definitions, pictures, and videos were presented contiguously to the EG; and the written form of the target words, audios, L2 translations, and L1 definitions were presented contiguously to the CG.

### Instruments

#### Questionnaire I

To assess participants’ receptive knowledge of the words, a questionnaire was adapted from the Vocabulary Knowledge Scale developed by Wesche and Paribakht (1996). The initial assessment of learner’s knowledge of the test items with a total of 48 words consisted of a receptive recall test in the form of English to Chinese translation. For each word, the English written form of the word was given, and the learners were required to declare if they have encountered that word before. If yes, its Chinese translation was asked.

#### Immediate and Delayed Post-tests

To evaluate participants’ performance after the treatment, an immediate post-test and a delayed post-test were developed by the researchers. In learning new vocabulary, establishing a memory link between the word form and its meaning is important (Nation, 2001; Laufer and Goldstein, 2004; Schmitt, 2000; Webb, 2015). Accordingly, the immediate post-test and the delayed post-test in this study assessed the creation of this form-meaning link. The written form of the target words was given on the left side of the test paper. The students were required to give the Chinese translation of these eight English words on the right side of the test paper within 2 min. The delayed post-test on word meaning was similar to the immediate post-test on word meaning, with the only difference being the target words were presented in an order different from that in the immediate post-test.

#### Questionnaire II and Interview

To address RQ3, questionnaire II and a semi-structured interview were developed. Questionnaire II was designed to investigate participants’ perceptions and attitudes toward the multimodal input method in vocabulary learning, which might help answer RQ 3. It is composed of 8 questions. This questionnaire was compiled with reference to Sydorenko (2010). Items 1–7 were measured in the form of a five-point Likert scale, ranging from 1 (strongly agree) to 5 (strongly disagree). Item 1 asked whether the multimodal input method motivated their interest in vocabulary learning. Items 2 to probed into which mode of the audios, videos, pictures, L1 translations, or combination of those modes helped most in retrieving the meaning of the target words in the immediate post-test. Question No. 7 explored students’ overall feelings toward the multimodal input method in vocabulary learning. Question No. 8 is an open question that queried participants’ suggestions on vocabulary learning. However, since none of the participants gave relevant feedback, the answers will not be analyzed and discussed in this study.

The interview in this study was designed to explore further what students think about the multimodal input method in vocabulary learning. This study adopted a semi-structured interview. Before conducting the interview, 5 questions were organized to collect students’ perceptions and attitudes toward the multimodal input approach. The five questions of this interview are as follows: (1) What methods do you usually use to learn vocabulary? (2) What do you think of learning vocabulary through its L2 translation, L1 definition, and audio pronunciation? (3) What do you think of learning vocabulary through its L2 translation, L1 definition, audio pronunciation, and pictures and videos? (4) Of these two methods, which one do you prefer? (5) Did the integration of L2 translation, L1 definition, audio pronunciation, and pictures and videos help you in this vocabulary meaning test? If yes, how? If not, why?

This interview started with the easiest question, as question 1 asked for information that is closely related to the students’ learning experience. Questions 2, 3, and 4 asked about interviewees’ attitudes toward the multimodal input approach and the monomodal input approach. Question 5 asked for interviewees’ perceptions toward the multimodal input approach. This interview was conducted by the researchers on the same day of the teaching phase when the time was convenient for the interviewees according to the interview outline. Responses on the interview were recorded, transcribed, and summarized for analysis.

It should be noted that both questionnaire II and the interview used students’ native language to ensure comprehensibility, and that the interview was conducted in the teachers’ office which was quiet and suitable for communication.

### Research Procedures

**FIGURE 2:**
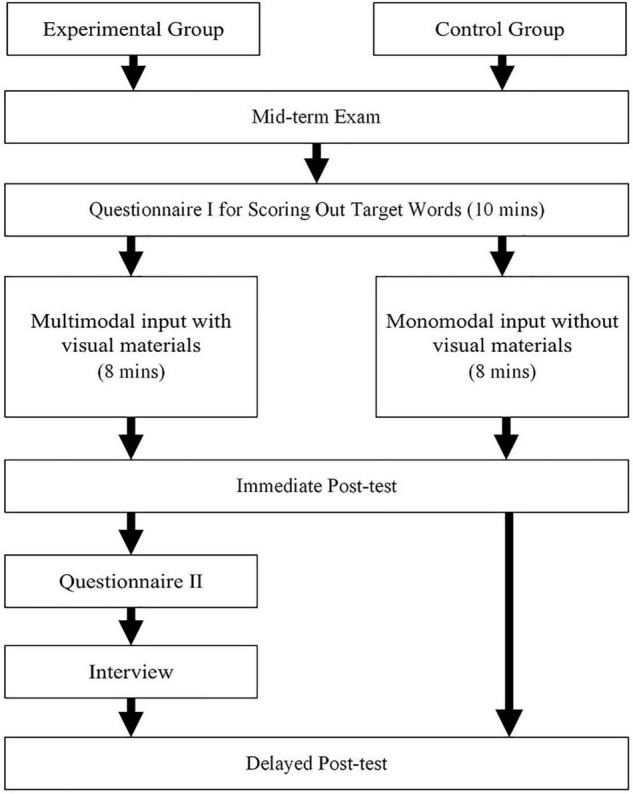
Experimental procedures.

This study took about 2 weeks to conduct and consisted of six phases. One week before the experiment, questionnaire I was handed out with the purpose of sorting out the target words. Following questionnaire I was the teaching phase, which was conducted 1 week later. Both groups learned word meanings through PowerPoint slides for a total of 8 min, with 1 min for each word and with the CG receiving monomodal input information (e.g., written form, L1 translation, L2 definition, and audio pronunciation) and the EG receiving multimodal input information (e.g., written form, L1 translation, L2 definition, audio pronunciation, and picture, video). After the teaching intervention, both groups were required to finish the immediate post-test within 2 min using a pen and paper. In addition, questionnaire II was distributed and the interview was conducted on the EG. One week later, a delayed post-test was conducted for both groups. Figure 2 shows the research procedures used.

### Data Collection and Analyses

In tackling RQ1, both immediate and delayed post-test scores were considered. In scoring the post-test and delayed post-test results for data analysis purposes, for each word, a learner received either 1 point or 0 points. A point was given for responses that were identical to its Chinese translation given on the slides. Responses with wrong Chinese translation and no response were counted as incorrect and received 0 points. Thus, the highest score is 8 and the lowest is 0. Both the immediate and delayed post-tests were marked by the same researcher, and the criterion of marking was the same. The scores were recorded by the researcher for data analysis.

The statistical data collected in this study are as follows: (1) mid-term scores of these two classes; (2) scores in the immediate post-test and those in the delayed post-test; (3) participants’ answers on questionnaire II. Besides, interviewees’ answers were recorded and transcribed with their permission, and were then summarized and analyzed to support statistical data and answer RQ 3.

An alpha level of *p* < 0.05 was set for all the tests. Effect size estimates were obtained by calculating *r* for the non-parametric tests and the independent samples *t*-test. Following Plonsky and Oswald (2014), *r*-values of 0.25, 0.40, and 0.60 were considered as small, medium, and large, respectively.

## Results

### Midterm Test of All Students From the Two Classes

At the very beginning of this study, in order to make sure that students in the two classes had the same level of English proficiency, an independent samples *t*-test was performed on the students’ mid-term English scores. Before conducting the *t*-test, the homogeneity of variance was checked by Levene’s test, and this assumption was met.

Table 2 presents the descriptive statistics of midterm test scores of students in the two classes.

**TABLE 2:** Independent samples *t*-test of midterm test scores of the two classes.

	N	Mean	*SD*	*Df*	*T*	*P*
EG	48	91.32	4.22	86	0.512	0.610
CG	40	90.85	4.40			

As shown in Table 2, the mean score of the CG is 90.85 and the mean score of the EG is 91.32. Table 2 shows that the significance of the mean score is 0.61, which is much greater than 0.05 and indicates no significant difference between the scores of the two groups. It is safe to say that the students in the two classes have the same level of English proficiency. This would presume that all the learners have an equal ability to learn L2 vocabulary and that, when given adequate instruction, they will all perform at the same level.

### Midterm Test of Students in the Experimental Group and the Control Group

In questionnaire I, eight target words were fixed, and the 28 students in the EG and 32 students in the CG declared their lack of knowledge of these eight target words. To once again render certain that the EG and the CG have the same level of English proficiency, another independent samples *t*-test was performed on the students’ mid-term English scores.

As shown in Table 3, the significance of the mean score is 0.928, which is much greater than.05 and indicates that there is no significant difference between the scores of the two classes. This shows that students in the EG and the CG have the same level of English proficiency.

**TABLE 3:** Independent samples *t*-test of midterm test scores in the experimental group (EG) and the control group (CG).

	N	Mean	*SD*	*Df*	*T*	*P*
EG	28	90.29	4.91	56	–0.091	0.928
CG	32	90.40	4.40			

### Immediate Post-test on Word Meaning

Regarding the effect of input, Table 4 displays the results of the immediate post-test of the 60 participants of the experiment. Each learner studied 8 words.

**TABLE 4:** Mann-Whitney *U*-test of immediate post-test in the EG and the CG.

	N	Mean	*SD*	*df*	*Z*	*P*	Effect size *r*
EG	28	7.14	1.24	58	0.033	0.974	0.004
CG	32	7.25	0.95				

Shapiro-Wilk tests were first conducted to determine the normality of the test scores of the EG and the CG. It showed that *p*_CG, immediate–post_ = 0 < 0.05, and that *p*_EG, immediate–post_ = 0 < 0.05, which meant that the immediate-test scores of the CG and the EG had a non-normal distribution. Thus, a Mann-Whitney *U*-test was performed.

Table 4 shows that learners in the CG obtained an average score of 7.25 out of 8 in the immediate post-test, which corresponds to a mean vocabulary learning rate of 90.6%, whereas the learners in the EG obtained an average score of 7.14 out of 8 in the immediate post-test, which corresponds to a mean vocabulary learning rate of 89.3%. As shown in Table 4, there is no significant difference in the mean score between the CG and the EG, with *z* = 0.033, *p* = 0.974 > 0.05, even though the mean score of the CG is slightly higher than that of the EG in the immediate post-test. The effect size for this difference was in the small range (*r* = 0.004).

### Delayed Post-test on Word Meaning

To explore the effectiveness of multimodal input in retaining the meaning of words compared with monomodal input, Shapiro-Wilk tests were first conducted to determine the normality of the score data of the EG and the CG. It showed that *p*_CG, delayed–post_ = 0.167, and that *p*_EG, delayed–post_ = 0.075, which meant that the delayed post-test scores of the CG and the EG had a normal distribution. An independent samples *t*-test was performed on delayed the post-test between the EG and the CG; the result of which is shown in Table 5.

**TABLE 5:** Independent samples *t*-test of delayed post-test in the EG and the CG.

	N	Mean	*SD*	*Df*	*T*	*P*	Effect size *r*
EG	28	3.57	1.93	58	0.954	0.344	0.123
CG	32	4.09	2.26				

As shown in Table 5, learners in the CG obtained an average score of 4.09 out of 8 in the delayed post-test, which corresponds to a mean vocabulary learning rate of 51.1%, whereas learners in the EG obtained an average score of 3.57 out of 8 in the delayed post-test, which corresponds to a mean vocabulary learning rate of 44.6%. Table 5 shows that there is no significant difference in the mean score on delayed post-tests between the EG and the CG, with *t* = 0.954, *p* = 0.344 > 0.05. Still, the mean score of the CG is slightly higher than that of the EG in the delayed post-test, and the effect size r = 0.123 falls into the small range.

Looking into the retention effect of multimodal input on learning of vocabulary meaning and its retention effect in comparison with the monomodal input method, Wilcoxon signed rank tests were then conducted. The results are shown in Table 6 below.

**TABLE 6:** Wilcoxon signed rank test comparison between the immediate and delayed post-tests.

	Immediate Post-test		Delayed Post-test		Z	P	Effect size *r*
		
	Mean	S.D.	Mean	S.D.			
EG	7.14	1.24	3.57	1.93	4.481	0.000	0.599
CG	7.24	0.95	4.09	2.26	4.639	0.000	0.580

Table 6 shows that there is a drop in the mean score of the EG and the CG from 7.14 in the immediate post-test to 3.57 in the delayed post-test and from 7.24 in the immediate post-test to 4.09 in the delayed post-test, respectively. The Wilcoxon signed-rank test of the EG shows a significant difference between the two post-tests, with a close-to-large effect size (*z* = 4.481, *p* = 0 < 0.05, *r* = 0.599). With regard to the CG, a significant difference between the two post-tests also emerged, with a close-to-large effect size (*z* = 4.639, *p* = 0 < 0.05, *r* = 0.58).

### Questionnaire II

Research question 3 in this study probed into participants’ attitudes toward the multimodal input method for learning vocabulary meaning. To address this question, students in the EG were required to respond to questionnaire II. Cronbach’s alpha of this questionnaire is 0.927, which means that the reliability of this questionnaire is sound.

As shown in Table 7, 57.1% of the participants strongly agreed and 28.6% of them agreed that the multimodal input method motivated their interest in learning vocabulary. Only 14.3% of the participants answered not sure, and none of them disagreed or strongly disagreed. It is noteworthy that majority of the students declared that the multimodality (the integration of AP, V, P, and L1L2) helped them most in retrieving the meaning of the target words in the immediate post-test. It is also worth mentioning that one student disagreed that pictures helped him or her in recalling the meaning of the target words in the immediate post-test. Based on question no. 7, 92.9% showed a positive attitude toward this teaching method.

**TABLE 7:** Results of questionnaire II.

Items	Strongly agree	Agree	Not sure	Disagree	Strongly disagree
1	16	8	4	0	0
2 (AP)	14	9	5	0	0
3 (V)	18	7	3	0	0
4 (P)	17	8	2	1	0
5 (L1L2)	15	6	7	0	0
6 (Multimodality)	19	7	2	0	0
7	17	9	2	0	0

*AP, audio pronunciation; V, video; P, picture; L1L2, L1 translation and L2 definition.*

### Interview

In order to support statistical data and to answer RQ 3, a semi-structured interview was conducted. The following is part of the interview in which 5 students (students A, B, C, D, and E) were selected randomly to participate after the immediate post-test, and shows some detailed information about students’ perceptions and attitudes toward the multimodal input method in learning vocabulary.

First, with regard to methods used by students to learn vocabulary, most of the students reported that they used applications to learn vocabulary, as they provided not only definitions and translations but also example sentences and audio. This indicates that the students generally show a favorable attitude toward online applications in vocabulary learning. What is more, student A took a negative attitude toward traditional paper dictionaries because he thought *it was too dull.* However, student D mentioned that he *used the application Leci* (a smart-phone app for English vocabulary learning) *as it provides L2 translations, L1 definitions, example sentences, and audio pronunciation, pictures, and videos*, but he usually does *not watch videos because it takes too much time*, which implied one of the shortcomings of the multimodal instructional materials during vocabulary learning. Second, as to opinions about the monomodal input method, most of the students said that it’s dull to learn vocabulary by this method, because it is unappealing and cannot leave a deep impression. This demonstrated the unique advantages of multimodal over unimodal learning materials, with the latter being judged to be *too dull* because they could leave only information of a single mode, the verbal one, in learners’ sensory memory. Third, with regard to opinions about the multimodal input method, most of the students commented on it rather favorably because videos attract them a lot. suggesting that the videos could be a little bit longer. Student E mentioned that with using video *it’s easier to memorize a word because it helps by visualizing.* However, one student showed a negative attitude toward the multimodal input method in vocabulary learning. He thought that videos in class interrupt the learning process. He did, however, show a positive attitude toward using pictures. Fourth, as to their preference between the multimodal input method and the monomodal input method, one student said that he liked the multimodal input method, and another student reported that both of them are nice. The other three students did not give any comment. Lastly, when asked which modes (L2 translation, L1 definition, audio pronunciation, and picture and video) helped them in retrieving words, two students answered “pictures,” because vivid pictures could attract their attention. One student answered pictures and audio because pictures deepened his memory of the word’s meaning, and audio helped him to learn the pronunciation, thus reinforcing memorization of the word. The other two students did not make any comment.

## Discussion

### Effects of Multimodal Input Method on Learning Vocabulary

Research question 1 of this study concerns the immediate and delayed effects of the multimodal input method on learning vocabulary and the method’s effect in comparison with that of the monomodal input method. First, by means of comparing self-reports on questionnaire I and the immediate post-test, the results indicate that the multimodal input method is effective and beneficial to students’ vocabulary learning. Second, compared with the monomodal input method, the statistic results of this study show that it is slightly less effective. As for the first result, it is in accordance with the findings of many previous studies that the multimodal input method is beneficial for vocabulary learning (Khezrlou et al., 2017; Peters, 2019; Puimège and Peters, 2019; Puimège and Peters, 2020). As for the second result, however, it seems inconsistent with the findings claimed by some previous studies, which concluded that students who learned with the multimodal input method either outperformed or showed no significant difference with those who did not (Sydorenko, 2010; Ramezanali and Faez, 2019; Alzahrani and Roberts, 2021). But the result is compatible with the finding of Acha’s (2009) in that children who process visual materials can be exposed to a higher cognitive load than those who only process the word and that children’s learning processes are hindered by limited working memory. The result is also in line with Boers et al.’s (2017) in that the provision of visual materials alongside textual information to elucidate the meaning of new words may reduce the amount of attention that L2 learners give to the words proper.

### How Does Multimodal Input Affect the Acquisition of Target Vocabulary

Research question 2 of this study probes into possible reasons that affected students’ immediate and delayed vocabulary learning and retention results in the EG. In this study, students in the EG were able to have sound vocabulary recall after being taught with the multimodal input method. as shown by immediate post-test. The results can be explained by referring to the advantages of multimodal input.

To begin with, multimodal materials used for the EG provided two retrieving cues for students. The materials for EG consisted of target words, L2 translations, L1 definitions, and audios. Besides, pictures and videos were utilized to illustrate the meaning of target words. Therefore, visual information and verbal information would enter sensory memory through the eyes and ears. Sensory memory allows for pictures, video, written forms of words, L2 translations, and L1 definitions to be held as visual images for a very brief time period in visual sensory memory and audio to be held as auditory images for a very brief time period in auditory sensory memory. Based on dual coding theory (Paivio, 1990), working memory temporally holds and manipulates knowledge in active consciousness, enabling students to construct referential connections between the two forms of mental representation (Mayer and Sims, 1994). As one student reported in the interview, the multimodal input method made it easier to memorize a word as it helped to visualize. Thus, in the post-tests, students in the EG had two retrieving cues for the meaning of target words and had sound vocabulary recall. Besides, the verbal and visual materials in this study were presented in an order of written form of word, audio, L2 translation, L1 definition, picture, and video, which means that these materials were presented contiguously, as referential connections are more easily built when both verbal and visual materials are presented contiguously (Mayer and Sims, 1994).

Despite the sound vocabulary recall that that EG had, the CG performed better in the two post-tests.

This result might be explained by the redundancy effect of cognitive load theory. The redundancy effect occurs when one source is sufficient to allow for understanding and learning while the other source merely reiterates the information of the first source in a different form. Abundant information was also recognized as a source of impairment in learner’s working processing capacity (Davis and Lyman-Hager, 1997). In this study, students in the EG had to divide their attention to different modes of information with a limited class time. On the positive side, the plentiful multimodal materials used in the EG, with the combination of verbal information and visual information, may aid memory trace in learners. However, the multimodal input method also forced the participants to handle additional visual information with a limited time, which increased their cognitive load. Besides, the provision of pictures alongside textual information to elucidate the meaning of new words may reduce the amount of attention that the students give to the word proper (Boers et al., 2017).

Another explanation might be found in the low cognitive ability of the students. The participants in this study were junior high school students, meaning that their cognitive ability is in the developmental stage. According to Plass et al. (2003), adults with low cognitive ability may learn worse when an additional picture is added in the multimodal input method of vocabulary learning. Compared with adults, the cognitive ability of junior high school students could be lower. It is, therefore, possible that elimination of some visual inputs or provision of additional input time could have led to a better result.

Lastly, the multimodal input method in class teaching was new to the EG, and it took time for students in this group to get used to this new type of input method, while students in the CG were familiar with the monomodal input method, as it is a conventional teaching method. Adding videos in class teaching reduced the amount of attention that students had to give to meaningful information. As one of the interviewees said, videos might interrupt class teaching. He also stated that adding a video might be helpful to learn vocabulary outside of class if a student has self-discipline. Junior high school students are easily attracted by videos themselves, which might actually hamper the process of mentally integrating visual information and verbal information on target words.

### L2 Learners’ Attitudes Toward the Multimodal Input Approach

Research question 3 of this study is answered by means of questionnaire II and interview. According to questionnaire II, most students in the EG adopted a favorable attitude toward the application of multimodal input in vocabulary learning. They thought this method motivated them in the process of vocabulary learning, which is similar to the questionnaire results discussed in the study of Sydorenko (2010). Most students declared that multimodalities of L2 translation, L1 definition, audio, picture, and video attracted their attention, deepened their impression of the words, and promoted their memory of the words. According to the interviews, the interviewees showed both favorable and critical attitudes toward the multimodal input method of vocabulary learning. Combined with the results of questionnaire II and the interview, the multimodal input of visual and verbal materials did help the students in arousing interests and understanding of the meaning of the new words, but at the same time, it brought the disadvantages of consuming time and distracting the attention of the students.

Although some studies (e.g., Taylor, 2005; Kam et al., 2020) have shown that learners do not necessarily have preferences for multimodal input, whose additional information may devolve as attention-getter and, thus, detrimental to vocabulary learning, positive perceptions of the learners benefiting from the advantages of multimodal input in this study have been previously identified by some scholars. Shadiev et al. (2020), for instance, developed a vocabulary learning system featuring image-to-text recognition (ITR) technology. Learners in the CG learned vocabulary through corresponding pictures in the textbook, while learners in the EG used the ITR learning system to learn vocabulary. Then, their perceptions were analyzed by a questionnaire survey and an interview. The quantitative results suggest that learners who study using the learning system featuring ITR technology learn vocabulary better than those who study using traditional methods. The qualitative results revealed that most learners in the EG had positive perceptions of this multimodal computer-assisted language learning (CALL) method. Bisson et al. (2015) also affirmed the crucial role of multimodal input in the acquisition of L2 vocabulary. In this study, participants who had been presented with a picture recalled significantly more L2 words after a week’s delay. In addition, the time spent looking at the pictures predicted recognition and recall scores. The results demonstrated the impact of exposure to multimodal input, especially the important role that pictorial information can play in L2 vocabulary acquisition.

The outcome of this research is that additional vivid visual information (picture and video) is useful for attracting learners’ attention and improving the learners’ learning motivation and satisfaction, thus inducing them to learn vocabulary more actively compared to other methods, particularly rote learning (Krashen, 1989; Pan, 2017; Blaz, 2018). In general, both the advantages and disadvantages of the multimodal input method of vocabulary learning are distinct to notice, as students can be influenced in either way. Students’ responses on both sides indicate that teachers should make efforts to increase the positive effects and avoid the negative effects of the multimodal input method in classroom teaching.

## Pedagogical Implications

In light of the reported findings, some implications for English vocabulary teaching and learning can be drawn based on the analysis of the results.

On the one hand, this study confirmed the great potential of multimodal input to bring significant increases in vocabulary learning. In addition, it also demonstrated that using different modes improves student’s perception of his or her own learning process; learners are more conscious and interested in learning materials. Hence, in order to promote learners’ vocabulary learning, teachers or instructional designers should try to use multimodal corpora like iWeb employed in this study, which provides an efficient approach to gain plentiful audiovisual materials in order to create a multimodal learning environment with informed use of visual and verbal information. On the other hand, this study indicates that compared with the effects of the monomodal input of vocabulary learning, the multimodal input showed less sound effect. It is suggested, thus, that practitioners should consider the processes involved in each task to impose the minimum cognitive load instead of mindlessly misusing such learning materials, especially under conditions where the wide availability of audiovisual input from the Internet and streaming platforms means that language learners can be easily overwhelmed by being exposed to large amounts of multimodal language input (Webb, 2015). In conclusion, even though the application of the multimodal input method is gaining attention and becoming popular in teaching, and using it is considered as a signal of keeping up with the trend (Zhang and Zou, 2021), the advantages and disadvantages should be critically analyzed before putting it into practice.

In addition, students need to be aware that human cognitive abilities are limited, so when they “have to” be exposed to large amounts of information in the classroom, they should allocate their attention appropriately according to their proficiency and modality preference (e.g., visual or auditory learner, etc.) in order to achieve more productive vocabulary learning outcomes.

Lastly, as highlighted in this article, cognitive load theory is of great value. It is the responsibility of instructional administrators to make teachers aware of the importance of taking it into account. Teaching vocabulary is not just a process of providing information: the more information provided is not always the better. If a teacher gives too much information to students simultaneously, the students will be at a risk of cognitive overload.

## Limitations and Suggestions

This study has a number of limitations.

First, the number of target words in this study is small, which has a direct impact on the application of the multimodal input method. Most studies reviewed in this study selected about 15 words or more. There are 17 target words in Perez et al. (2014), 18 target words in Zarei and Khazaie (2011), and as many as 82 target words in Chun and Plass (1996a; 1996b). To improve the generalizability of the findings of this study, future studies should expand the number of target words.

Second, this study only targeted Grade 9 students in a single school. Moreover, the EG and the CG were the top students of this school; generally, “results must be viewed in terms of the level of the learner’s L2 language ability and cannot be generalized to all learners” (Chun, 2006, p. 77). That is to say, the differences of students should be taken into consideration. Future studies can further explore the effects of learner-related factors, such as different English proficiency levels, learning styles, and levels of motivation.

Lastly, the instruments used in this study are also limited. This study conducted two post-tests to explore the effects of the multimodal input method of vocabulary learning. The questionnaire and interview were then used to explain the results. In a future study, eye-tracking could be a valuable research instrument, as it produces a real-time record of learners’ eye movements during tasks, and provides researchers with information not only about what students are paying attention to but also about how long they are focusing on a certain word or picture (Kang et al., 2020).

The results of this study and major findings should be interpreted in light of the aforementioned limitations.

## Conclusion

This study explored issues surrounding the multimodal input of verbal and visual materials and monomodal input of verbal materials in English vocabulary learning. This research proved that the multimodal input method has a positive effect on vocabulary learning. However, compared with the monomodal input method applied in this study, this method showed fewer sound effects on recalling vocabulary meaning in the longer run. The redundancy effect of cognitive load might have affected the results of this study. Additional visual information forced the students to handle additional information in a limited time, which increased their cognitive load. With regard to students’ attitudes toward the application of the multimodal input method of vocabulary learning, there were both positive and negative opinions. On the one hand, they reported that this method motivated their interest, deepened their impression of the words, and promoted their memory of the words. On the other hand, this method can be time-consuming and distract students’ attention.

In general, the results of this study indicate a promising application of the multimodal input method in junior high school students’ vocabulary learning. Reasons contributing to the less sound effect of the multimodal input method compared with the monomodal input method and students’ opinions on the advantages and disadvantages of this method are of referential significance in maximizing its strengths and minimizing its weaknesses.

## Data Availability Statement

The raw data supporting the conclusions of this article will be made available by the authors, without undue reservation.

## Ethics Statement

The studies involving human participants were reviewed and approved by the Ethics Committee of School of Foreign Studies, South China Normal University. Written informed consent to participate in this study was provided by the participants’ legal guardian/next of kin.

## Author Contributions

XL: conceptualization, supervision, project administration, and funding acquisition. WL and XL: methodology, investigation, data curation, and writing—original draft preparation. JY and ZZ: writing—review and editing. WL: visualization. All authors have read and agreed to the published version of the manuscript.

## Conflict of Interest

The authors declare that the research was conducted in the absence of any commercial or financial relationships that could be construed as a potential conflict of interest.

## Publisher’s Note

All claims expressed in this article are solely those of the authors and do not necessarily represent those of their affiliated organizations, or those of the publisher, the editors and the reviewers. Any product that may be evaluated in this article, or claim that may be made by its manufacturer, is not guaranteed or endorsed by the publisher.
